# Discussion on Static Resistance of Granite under Penetration

**DOI:** 10.3390/ma16093353

**Published:** 2023-04-25

**Authors:** Xiaodong Nie, Xiangyun Wu, Zhi Yi, Zhilin Long, Hui Zhou, Nan Ji

**Affiliations:** 1 School of Mechanical Engineering and Mechanics, Xiangtan University, Xiangtan 411105, China; m13170328536@163.com (X.N.); longzl@xtu.edu.cn (Z.L.); 16637706172@163.com (N.J.); 2 Institute of National Defense Engineering, Academy of Military Sciences, People’s Liberation Army, Luoyang 471023, China; freeyogism@163.com; 3 State Key Laboratory of Mining Response and Disaster Prevention and Control in Deep Coal Mine, Anhui University of Science and Technology, Huainan 232001, China; huizhou9509@163.com

**Keywords:** penetration resistance, granite, simulation, RHT model

## Abstract

A total of 9 tests were carried out with 30 mm and 78 mm caliber scaled projectiles penetrating into granite targets. The penetration depth, crater diameter, and mass loss rate were examined and discussed. The results indicate that the dimensionless penetration depth of large-caliber projectiles is 20% greater than small-caliber projectiles. Based on the description of static resistance *R_a_* in the Forrestal semi-empirical formula, the size effect of dimensionless penetration depth can be attributed to the size effect of static resistance *R_a_*, and it can be seen that the penetration static resistance of projectile A is 40% higher than that of projectile B. Numerical simulations of projectile penetration into granite targets were conducted using the finite element program ANSYS/LS-DYNA. In terms of penetration depth and crater damage, the numerical simulation results agree well with the test data. This suggests that the selection of parameters was reasonable. The influence of compressive strength, projectile striking velocity, mass, diameter, and caliber–radius–head (CRH) ratio on the static resistance *R_a_* were studied by RHT model parameterization. Based on the numerical results from the parametric study, an empirical formula was derived to predict the static resistance *R_a_*.

## 1. Introduction

As common engineering materials, concrete and rock are often used in protective engineering against the attack of weapons. Hence, a systematic study on the mechanisms of these materials against projectile penetration is of great significance to enhance the anti-penetration ability of military protection engineering and ensure the safety of lives. At present, there exists a large amount of research on projectile penetration into concrete [1,2,3]. These studies indicate that compressive strength is an important factor influencing penetration resistance performance, and for rock materials, their microstructure has a significant impact on uniaxial compressive strength [4]. The research in Ref. [4] also shows that there is a strong correlation between the uniaxial compressive strength of rocks and their Brazilian tensile strength and point load strength. It is difficult to carry out experimental research on projectile penetration into rock materials, resulting in very limited research on projectile penetration into rock materials. The penetration resistance performance of normal strength concrete, different types of UHPC (ultra-high performance concrete), and granite targets were studied by Zhang et al. [5]. Through the test, it was found that under the same penetration conditions, the penetration depth of the granite target was lower than the normal strength concrete and the ultra-high performance concrete target. Zhang [6] investigated the penetration resistance performance of 150 MPa granite target against two series of reduce-scaled ogive-nosed projectile penetrations, and the tests showed that under the same striking velocity, the penetration depth of granite was significantly lower than that of ordinary concrete. The penetration resistance performance of 120 MPa limestone against high-velocity projectile penetrations with striking velocities from 760 to 1450 m/s was studied by Shen et al. [7]. Moreover, an empirical formula has been developed to estimate the depth of penetration into limestone. Most of the above studies are based on small-caliber projectile tests, and there are limitations to extrapolating data from small-scale tests to full-scale applications.

To generalize the research results of small-scale experiments to full-scale scenarios, it is essential to determine whether the law of replication scaling holds. Most of the research on the penetration scaling law mainly focuses on concrete materials, and the question of whether the scaling law applies to penetrations remains a topic of controversy. Frew [8] investigated the penetration depths in 58 MPa concretes by two similarly shaped projectiles with different dimensions, and found that the dimensionless penetration depth of the two groups of tests is almost the same. The research in Refs. [9,10] shows that the coarse aggregates with a fixed size (not scaled-down replicas) can explain the size effect in the depth of penetration. Wu [11] conducted projectile penetration tests with six different scales to reveal the influencing mechanism of the size effect. In the test, it is found that the penetration depth has an obvious size effect, and the size effect can be attributed to strain-rate effects. The Forrestal semi-empirical formula [12] is based on the theory of dynamic cavity expansion. It established an expression about penetration resistance and believed that the static resistance *R_a_* is only related to the unconfined compressive strength of the concrete. In addition, Forrestal obtained an empirical formula for calculating the static resistance *R_a_* through small-scale projectile (12.9 mm < d < 30.5 mm) penetration tests. However, in his subsequent studies on larger projectiles (d = 76.2 mm) [13,14], it was found that the calculated static resistance value using the empirical formula was much larger than the experimental value. Other researchers [8,15] have also provided different empirical formulas, but their scope of application is also very limited.

Due to time and economic constraints, full-scale tests are rarely feasible when studying penetration problems. The numerical simulation method provides an alternative for studying the penetration problem. In the research of Liu [16], the finite element program LS-DYNA is used to examine the penetration resistance performance of UHPC targets with unconfined compressive strengths ranging from 90 MPa to 190 MPa. The targets were subjected to projectile penetration at velocities between 300 m/s and 1000 m/s. In their study, Yang [17] employed the Riedel–Hiermaier–Thoma (Mat_RHT) model to clarify the penetration resistance performance of 35 MPa concrete when subjected to projectile penetration. The results suggest that at low-impact speeds (about <600 m/s), the dynamic resistance is influenced by both the compressive strength and initial impact velocity. At present, numerous numerical studies have been carried out to simulate the penetration of projectiles into concrete. In contrast, there has been minimal numerical research investigating the impact response of rock under high-velocity projectile penetration.

**Figure 1:**
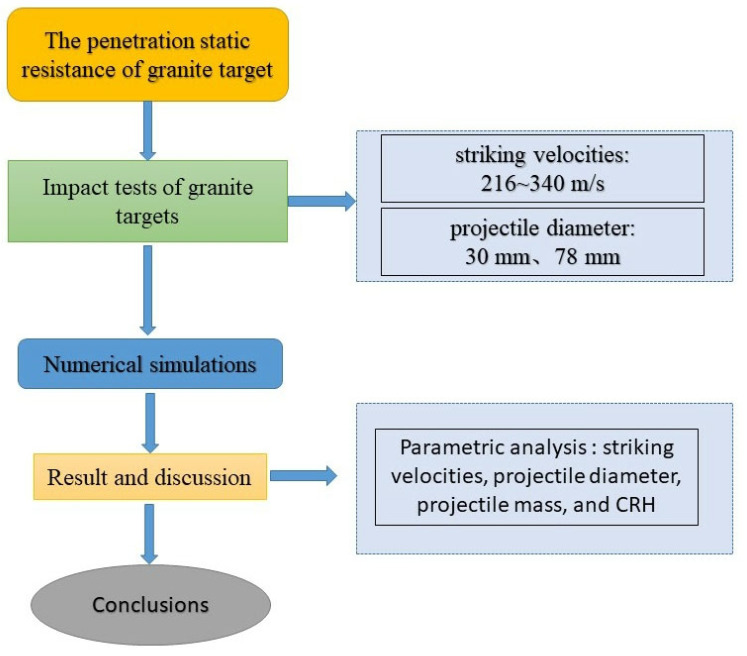
Flow chart for methodology.

In this paper, an experiment was conducted on a granite target subjected to 2 series of ogive-nosed projectile penetrations at impact velocities ranging from 216 m/s to 340 m/s. The depth of penetration (DOP) was compared and discussed. Based on the description of static resistance *R_a_* in the Forrestal [12] semi-empirical formula, the size effect of dimensionless penetration depth can be attributed to the size effect of static resistance *R_a_*. Furthermore, the finite element program LS-DYNA was used to simulate the penetration process on granite targets. According to the results of numerical simulation, the empirical formula for calculating the penetration static resistance is derived. The empirical formula proposed in this paper not only considers the compressive strength of granite materials but also comprehensively considers the influence of striking velocities, projectile diameter, projectile mass, and CRH on the penetration static resistance, making it applicable to a wider range of scenarios. A flow chart for the methodology of this paper is provided in Figure 1.

## 2. Research Methodology

### 2.1. Projectile

**Figure 2:**
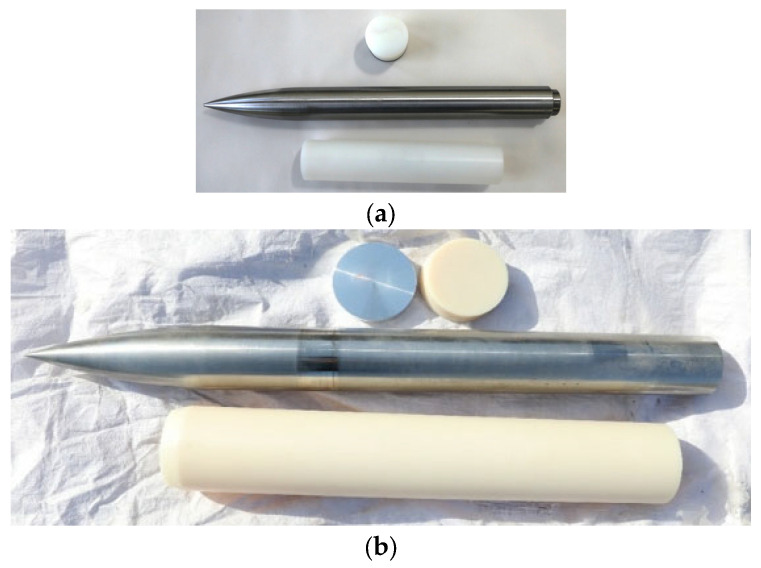
Ogive-nosed projectiles: (**a**) projectile A; (**b**) projectile B.

The penetration tests were conducted with two series of ogive-nosed projectiles (A and B). The projectiles were fabricated using 35CrMnSiA steel rods with a yield strength of 1300 MPa and the shapes of the projectiles are shown in Figure 2. The basic parameters of the projectiles are shown in Table 1. The CRH (caliber–radius–head ratio) is an important design parameter for projectiles, which refers to the ratio between the caliber of the projectiles and the radius of its ogive nose.

### 2.2. Granite Targets

**Figure 3:**
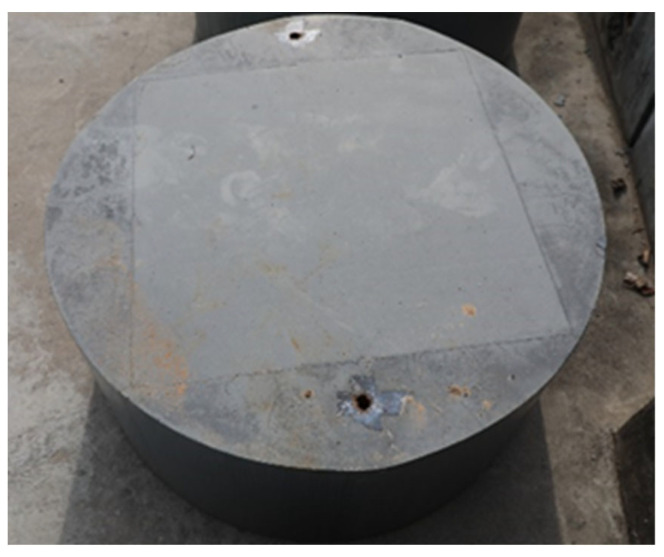
Granite targets.

Granite, as a natural protective material for underground structures, has good protective ability against weapons and has been widely used in various protection projects. The penetration test results are greatly affected by the free surface effect of the target in Zhang’s [6] experimental study. A study by Xue [18] showed that when the thickness of the steel hoop is 5 mm and the striking velocity is lower than 800 m/s, the impact of the transverse free-surface effect can be ignored if the ratio of projectile to target diameter is greater than 15. Taking into account that the maximum diameter of the projectile is 78 mm, to ensure a diameter ratio of more than 15 between the projectile and the target, the diameter of the target plate should not be less than 1170 mm. Therefore, the dimensions of the granite targets used in the experiment were as follows: 1300 mm in diameter and 600 mm in thickness. The granite was placed at the center of a 5 mm thick steel hoop with its surrounding filled with concrete. This procedure was also adopted by refs. [5,19]. These targets were designed to be the same size as shown in Figure 3. According to the test method of Chinese Standard GB/T 23561.7-2009, uniaxial compression and uniaxial tension experiments were performed on the granite specimens, and the obtained mechanical parameters of the granite are shown in Table 2.

### 2.3. Test Set-Up

**Figure 4:**
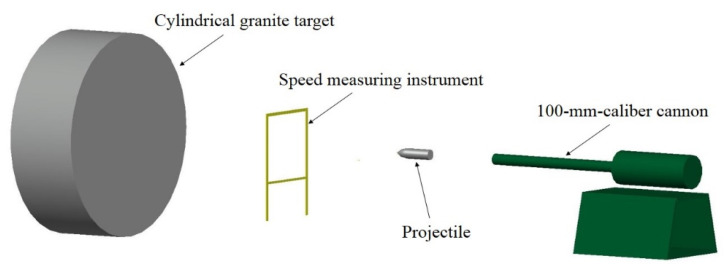
Schematic diagram of the projectile penetration tests.

**Figure 5:**
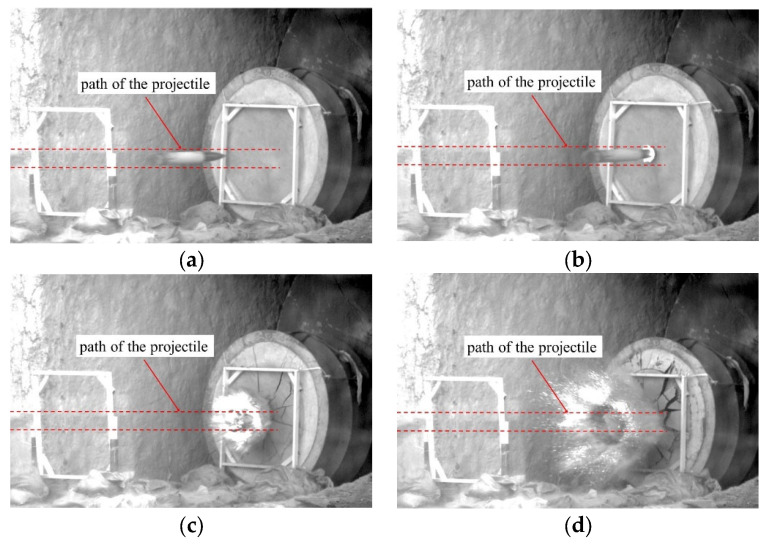
Photographs of typical projectile penetration into granite targets: (**a**) 0 ms; (**b**) 2 ms; (**c**) 4 ms; and (**d**) 8 ms.

The 35 mm caliber smooth-bore powder gun was utilized to launch projectile A. The 100 mm caliber cannon was used to launch projectile B, which is larger than projectile A. In order to meet the test requirements, sub-caliber launch technology was utilized because the diameters of the projectiles were smaller than the inner diameter of the gun. In order to fit tightly into the gun bore, nylon sabots and obturators were fitted to the projectiles. If the distance between the gun and the target is too large, the projectile may produce an attack angle before hitting the target, which will have a significant impact on the penetration results. Therefore, in this paper, the distance between the gun and the target is set to 10 m. Figure 4 displays the schematic diagram of the projectile penetration process. The study presented in Ref. [6] shows that when a projectile penetrates granite at impact velocities below 350 m/s, there is no significant deformation of the projectile during the penetration process. Therefore, the projectile velocities in this paper were all below 350 m/s. To measure the striking velocity, a pair of metal wire nets was positioned in the straight trajectory of the projectiles between the target and the smooth-bore powder gun. Figure 5 displays photographs captured by the high-speed camera system at the moment when projectiles collided with the target. These photographs can help us determine the projectile’s flight attitude before hitting the target.

### 2.4. Results and Analysis

**Figure 6:**
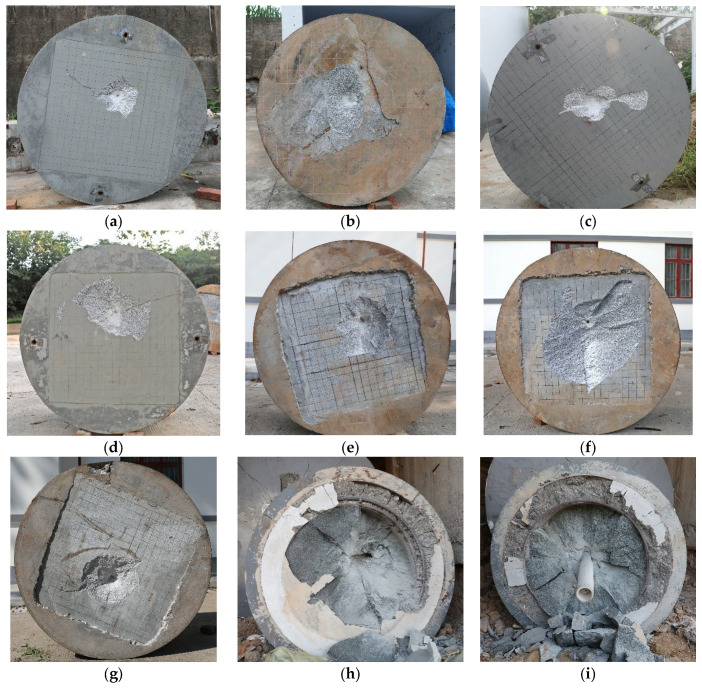
Localized damages of targets: (**a**) gr-1; (**b**) gr-2; (**c**) gr-3; (**d**) gr-4; (**e**) gr-5; (**f**) gr-6; (**g**) gr-7; (**h**) gr-8; and (**i**) gr-9.

**Figure 7:**
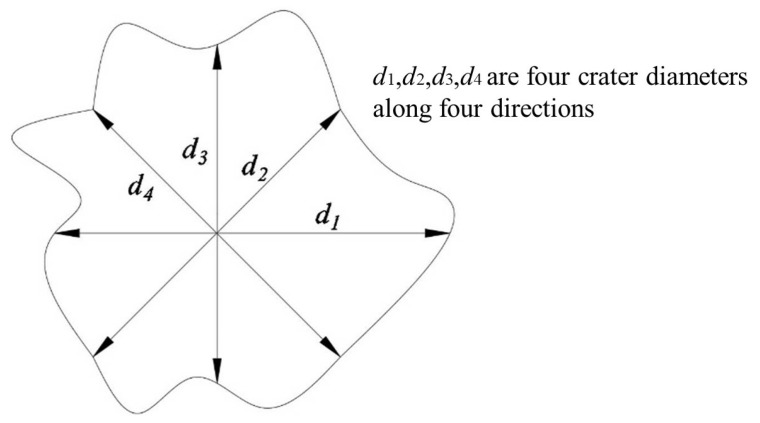
Measurement of average crater diameter.

In Figure 6, we show the impacted surfaces and their corresponding damage in each test. Table 3 shows the depth of penetration (DOP), static resistance, mass loss rate, and crater diameter (dc). An average of four diameters along four directions was used to calculate the crater diameter on granite targets, which is shown in Figure 7.

**Figure 8:**
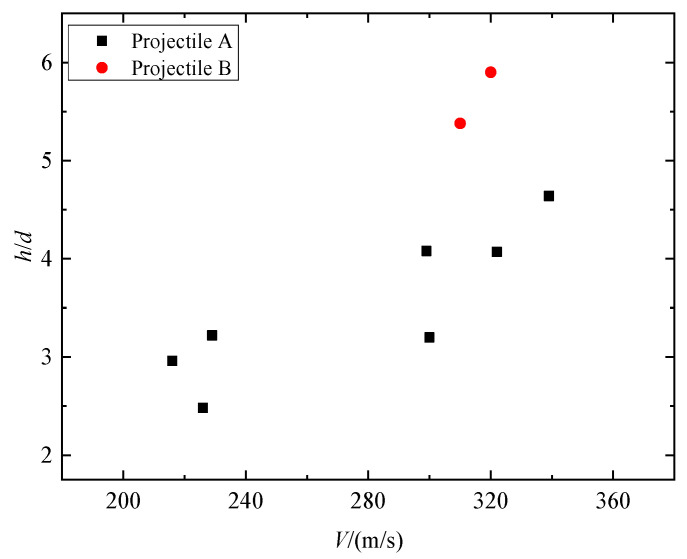
Dimensionless DOP versus the striking velocities of projectiles.

Figure 8 shows impact test results regarding dimensionless penetration depth at striking velocities of 216~340 m/s. From the observation, it can be inferred that the depth of penetration displayed a tendency to rise as the impact velocities increased. Taking Tests 1–7 for example, when the striking velocity continually increased from 216 m/s to 340 m/s, the DOP gradually increased from 89 mm to 139 mm. At the impact velocity of 216~340 m/s, the dimensionless DOP for projectile B was larger than that of projectile A. This shows that the penetration depth of the granite target has a size effect. According to Forrestal [12], research on concrete penetration and penetration depth is closely related to penetration resistance. This means that the size effect of penetration depth can be attributed to the size effect of penetration static resistance. The penetration static resistance *R_a_* obtained from the test in Table 3 can also confirm this point; at the striking velocity of 300 m/s, the penetration static resistance of projectile A is 40% higher than that of projectile B.

**Figure 9:**
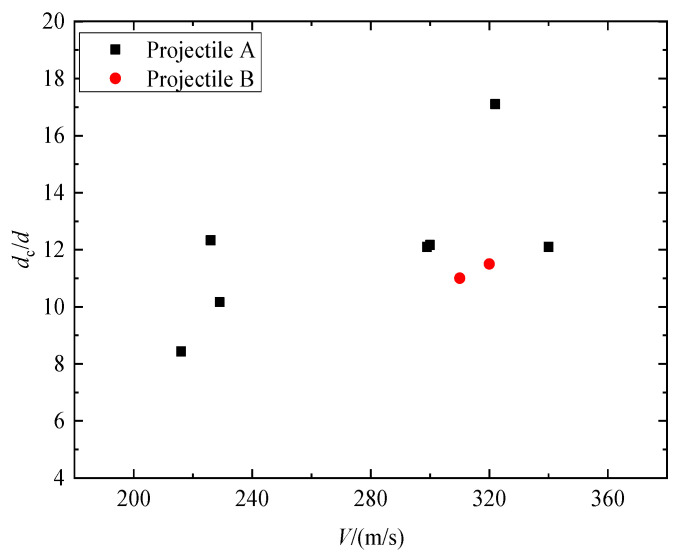
The crater diameter versus the striking velocities of projectiles.

Figure 9 illustrates the relationship between the striking velocities ranging from 216 m/s to 340 m/s and the resulting crater diameter of the granite target. It can be seen that with an increase in projectile impact velocity, the diameter of the crater increased. At the same speed, the dimensionless crater diameters of the two series projectiles are similar, and it can be considered that the crater diameter meets the penetration similarity rate.

**Figure 10:**
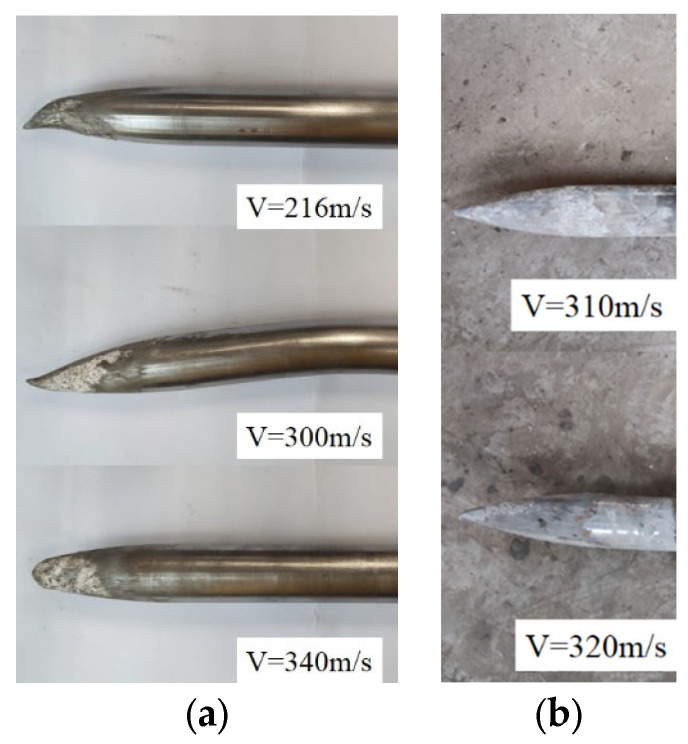
The projectile abrasions and damages: (**a**) projectile A; (**b**) projectile B.

Figure 10 shows the projectile head and surface damage and abrasions after impact testing. Observed on the projectile B head, there is no visible damage or deformation, but for projectile A, the projectile head suffered some minor abrasions, and the abrasions degree increases with the increase in striking velocities.

**Figure 11:**
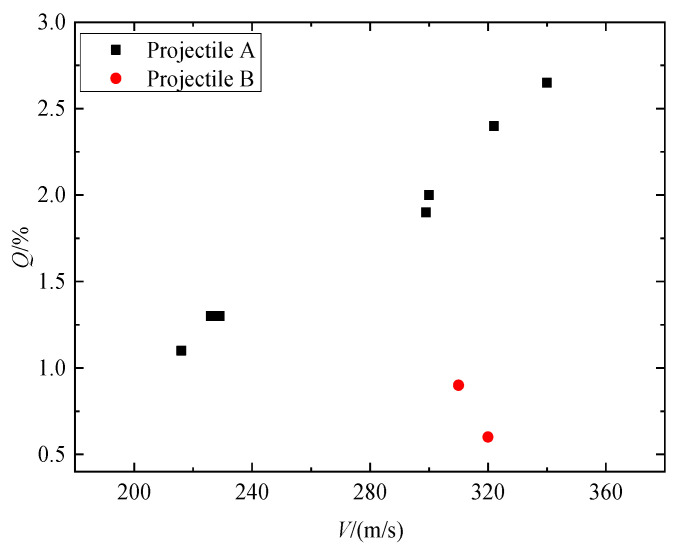
The mass loss rate of the projectile versus striking velocities.

Figure 11 illustrates the mass loss rate *Q* of the two series of projectiles. It is clear that as striking velocities increase, the mass loss rate of the projectile increases continuously. At similar striking velocities, the mass loss rate of projectile B is much lower than that of projectile A. This shows that the mass loss rate of the projectile has a size effect. Shen [7] studied the mass loss of projectiles penetrating limestone and believes that the main reason for the mass loss of projectiles is the strong friction between the projectile and the target. It is generally believed that the greater the penetration resistance, the greater the corresponding friction between the projectile and the target. This means that the size effect of the projectile mass loss rate can be attributed to the size effect of penetration static resistance.

## 3. Numerical Simulations

**Figure 12:**
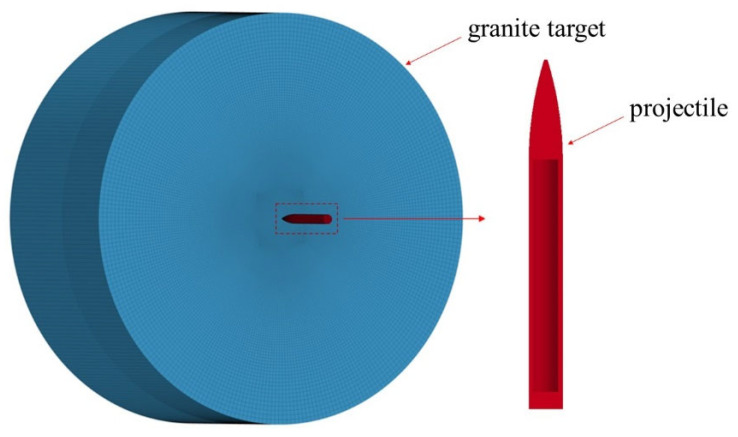
Finite element model for projectile-impact granite target.

A numerical survey is carried out in this section to simulate projectile penetrating into granite targets with the finite element program ANSYS/LS-DYNA. In the numerical simulation, granite targets and projectiles are the same dimensions as in the tests described above. The finite element model for the projectile impact granite target is shown in Figure 12. Eight-node solid elements, SOLID164, were used for the projectile and UHPC target. In LS-DYNA, the time step can be controlled by the keyword *CONTROL_TIMESTEP, which is multiplied by a factor of 0.9 for general problems and a factor of 0.67 for penetration and explosion problems by default. Referring to the convergence test in Ref. [20], and further verified in this paper with three different element sizes of 1 mm, 2 mm, and 4 mm, a fine element size of 2 mm is used within the diameter range of 0.4 m in the center of the target, and the rest of the section is modeled with a coarse element size of 4 mm. The element size of the projectile is set to 1.5 mm. The interface between granite targets and projectiles is composed of *CONTACT_ERODING_SURFACE_TO_SURFACE. The longitudinal displacement of the target is constrained in the numerical model. Since the diameter of the target is 15 times larger than that of the projectile [18], the numerical results are not affected by the transverse boundary conditions of the target. The keyword *INITIAL_VELOCITY_GENERATION is used in the numerical model to apply the initial velocity to simulate the launch of the projectile.

### 3.1. Material Models

In LS-DYNA, there are several models available to simulate projectile penetration into granite targets, including Johnson Holmquist Concrete (Mat_HJC), Riedal–Hiermaier–Thoma (Mat_RHT), and Concrete Damage Rel3 (Mat_72 Rel3). In the current simulation, granite targets are modeled using RHT [21]. This material model can well simulate the deformation and fracture characteristics of rock materials under extreme loads such as explosion and impact. It is worth mentioning that the RHT model is constructed using conventional concrete as the basis, and it was implied that the existing RHT parameters proposed for normal concrete might not be applicable to granite.

In the RHT model, The RHT model is mainly composed of the state equation and the constitutive equation. Under high-stress conditions, the strength of the material can be ignored compared with the pressure it receives. At this time, the pressure in the material needs to be calculated through the state equation. The pressure is described by a Mie–Gruneisen form with a polynomial Hugoniot curve. The EOS is expressed as:

(1) $$p\left(\rho ,e\right)=\frac{1}{\alpha }\left\{ \begin{array}{l} \left(B_{1}+B_{2}\eta \right)\alpha \rho e+A_{1}\eta +A_{2}\eta^{2}+A_{3}\eta^{3}\;\;\;\;\;\;\;\eta >0\\ B_{1}\alpha \rho e+T_{1}\eta +T_{2}\eta^{2}\;\;\;\;\;\;\;\;\;\;\;\;\;\;\;\;\;\;\;\;\;\;\;\;\;\;\;\;\;\;\;\;\;\;\;\;\;\eta <0 \end{array}\right.$$

where *A*_1_, *A*_2_, and *A*_3_ are the hugoniot polynomial coefficients, *B*_1_, *B*_2_, *T*_1_, *T*_2_ are the parameters for polynomial EOS, $\alpha_{0}$ is the initial porosity, and ρ is the density.

The RHT model is divided into three stages: elastic stage, linear hardening stage, and damage softening stage, which correspond to the following equations:

The elastic–plastic yield surface for the RHT model is given by:

(2) $$\sigma_{elastic}^{*}\left(p,\theta ,\dot{\varepsilon }\right)=\sigma_{TXC}^{*}\left(p_{s,el}\right)\cdot R_{3}\left(\theta \right)\cdot F_{rate}\left(\dot{\varepsilon }\right)$$

where $\sigma_{elastic}^{*}\left(p,\theta ,\dot{\varepsilon }\right)$ is the normalized strength relative to the compressive strength, $R_{3}\left(\theta \right)$ is the Lode angle factor, and $F_{rate}\left(\dot{\varepsilon }\right)$ is the strain rate strength factor.

The linear hardening stage is given by:

(3) $$\sigma_{Yhaard}=\sigma_{elastic}+3G\xi \varepsilon_{p}$$

where *G* is the shear modulus and ξ is a reduction factor representing the hardening in the model.

The resulting damaged surface is given as:

(4) $$\sigma_{damage}=\left(1-D\right)\sigma_{fail}+D\sigma_{residual}$$

(5) $$\sigma_{residual}=A_{f}\times {\left(p^{*}\right)}^{n_{f}}$$

where $\sigma_{damage}$ is the equivalent stress in the damage stage, $\sigma_{residual}$ is the residual equivalent stress, $A_{f}$, $n_{f}$ are the residual surface parameters, and *D* is the damage parameter.

Accurate prediction of granite target responses to projectile penetration requires parameter calibration in the RHT model. Some parameters of the RHT model are determined by quasi-static mechanical tests, with a density *ρ* = 2450 kg/m^3^, compressive strength *f*_c_ = 160 MPa, and elastic modulus *E* = 44 GPa, Poisson’s ratio υ = 0.19, shear modulus *G* = 18.5 GPa, relative tensile strength $f_{t}^{*}=0.061$, relative shear strength $f_{s}^{*}=0.267$, *A*_1_, *A*_2_, and *A*_3_ are the polynomial coefficients, and according to the description of the impact adiabatic equation in Ref. [22], *A*_1_ = *ρc*^2^ = 77.2 GPa, *A*_2_ = *ρc*^2^(2*s* − 1) = 56.5 GPa, *A*_3_ = *ρc*^2^(3*s*^2^ − 4*s* + 11) = 16.5 GPa, *T*_1_, *T*_2_ are the parameters for polynomial EOS, and *T*_1_ = *ρc*^2^ = *A*_1_ = 77.2 GPa, *T*_2_ = 0. According to the analysis results in Ref. [23], the crush pressure is taken as *p*_el_ = *f*_c_/3 = 35.3 GPa. Other parameters were fine-tuned based on the parameters in Refs. [17,21,22]. The RHT model parameters for granite are listed in Table 4. In addition, to prevent distortion of elements during simulation, The MAT_ADD_EROSION was employed in the material model to remove elements that exhibit significant distortions. In the research in Ref. [24], both shear strains and maximum principal strains of 0.13 were adopted to numerically study the impact response of G-UHPC targets. Ref. [3] chose tensile and shear strains as the erosion criteria, and the value are all set as 0.2 for the UHPC. In this study, erosion criteria with maximum principal strains of 0.28 were adopted to numerically study the penetration resistance performance of granite targets.

MAT_PLASTIC_KINEMATIC is commonly used to describe the plastic behavior of metals, and the model can be represented as follows:

(6) $$\sigma_{Y}=\left[1+{\left(\frac{\dot{\varepsilon }}{C}\right)}^{1/P}\right]\left(\sigma_{0}+\beta E_{P}\varepsilon_{eff}^{p}\right)$$

where $\sigma_{Y}$ is the yield strength; $E_{P}$ is the plastic hardening modulus, $E_{P}=E_{t}E/\left(E-E_{t}\right)$; $\varepsilon_{eff}^{p}$ is the effective plastic strain; β is the hardening parameter; and *C*,*P* are the strain rate parameters.

In the current simulation, MAT_PLASTIC_KINEMATIC was used for the projectile, and the model parameters for the projectile are listed in Table 5.

### 3.2. Modeling Results

Table 6 shows comparisons between experiment and numerical simulation results of DOP for granite targets. It can be seen that all the absolute deviations of numerical results fall within a reasonable range when predicting DOP of granite targets at impact velocities of 216 m/s, 300 m/s and 340 m/s. Therefore, numerical simulation is highly capable of accurately predicting the DOP of a projectile when penetrating granite targets.

**Figure 13:**
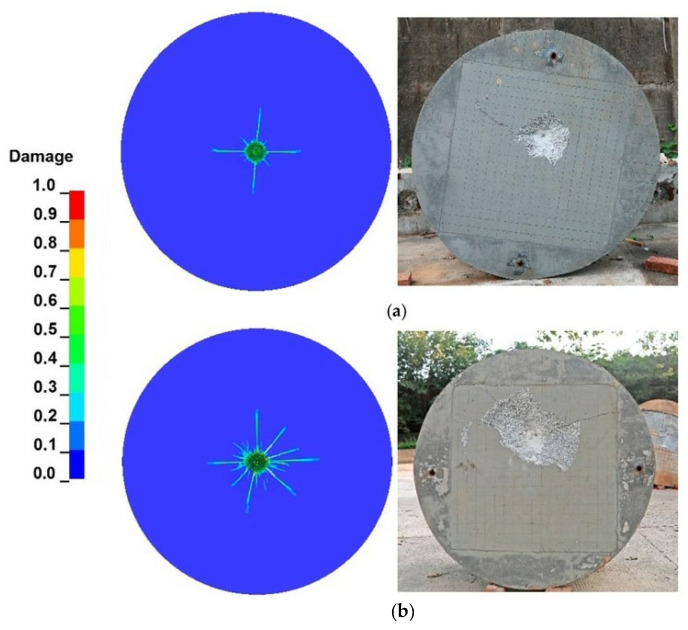

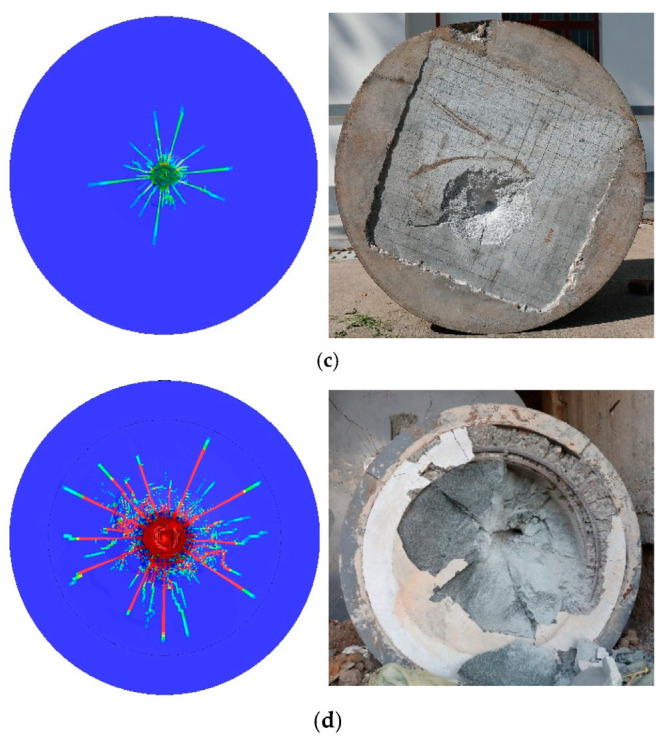
Crater diameters of granite targets: (**a**) gr−1; (**b**) gr−4; (**c**) gr−7; and (**d**) gr−8.

The damage factor *D* is commonly used in numerical simulations under the RHT model to reflect the damage caused by projectiles when penetrating granite targets. The definition of damage parameter *D* is the ratio of the accumulated equivalent plastic strain increment to the final failure equivalent plastic strain during the loading process of the material, and $0\leq D=\sum \left(\Delta \varepsilon_{p}/\varepsilon_{p}^{fail}\right)\leq 1$. When *D* = 0, the material is considered intact; when *D* = 1, the material is considered completely damaged. Figure 13 illustrates the impact-induced surface damage of a granite target when struck by projectile A and projectile B. It is evident that the numerical simulation results show remarkable conformity with the experimental data. The crater diameter trend with respect to impact velocity derived from numerical simulations agrees well with the experimental results.

## 4. Discussion

Forrestal [12] obtained the method for calculating the penetration static resistance *R_a_* based on the cavity expansion theory:

(7) $$R_{a}=\frac{N^{*}\rho_{t}V^{2}}{\left(1+\frac{\pi d^{3}N^{*}\rho_{t}}{2m}\right)\exp\left[\frac{\pi d^{2}\left(h-2d\right)N^{*}\rho_{t}}{2m}\right]-1}$$

where *R_a_* is the static target resistance; $\rho_{t}$ is the target density; *d* is the projectile diameter; *m* is the projectile mass; *V* is the projectile velocity; *h* is the penetration depth; and N^*^ is the ogive nose shape factor, and it depends on the projectile CRH:

(8) $$N^{*}=\frac{8CRH-1}{24CRH^{2}}$$

According to Forrestal research, *R_a_* depends only on the unconfined compression strength of concrete and can be modeled as: $R_{a}=82.6f_{c}^{0.456}$; Frew [8] fitted the penetration test data of 58 MPa concrete and believes that $R_{a}=72.0f_{c}^{0.5}$; and Wu [15] obtained the *R_a_* expression for high-strength concrete targets impacted by projectiles and believes that $R_{a}=127.7f_{c}^{0.325}$. It can be seen that the value of *R_a_* varies significantly for different target materials. Cheng [25] believes that the static resistance is significantly related to the penetration speed based on the theoretical analysis and experiment, and it cannot be simply be considered that static resistance is only related to the unconfined compressive strength. This paper assumes that the static resistance has the following form:

(9) $$R_{a}=A\cdot m^{\alpha }\cdot d^{\beta }\cdot CRH^{\gamma }\cdot f_{c}^{\lambda }\cdot V^{\xi }$$

A numerical simulation involving 28 projectiles was conducted on granite targets to analyze the impact of uniaxial compressive strength of granite, striking velocity, projectile mass, CRH, and diameter on static resistance values. The setup of the parametric studies and the corresponding static resistance values are given in Table 7.

### 4.1. Effect of Striking Velocity

**Figure 14:**
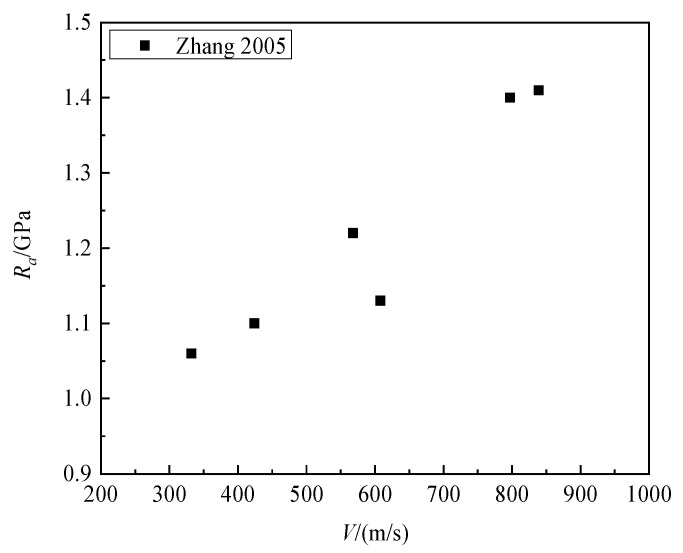
*R_a_* versus impact velocity for granite [6].

In earlier studies, it was commonly believed that the static resistance force was independent of velocity [8,12]. However, research in Ref. [25] showed that the static resistance *R_a_* is correlated with impact velocity. Figure 14 presents the variation in granite static resistance at different impact velocities in Ref. [6], indicating a significant increase in static resistance with the increase in impact velocity.

Yang [26] found through numerical simulations that when the impact velocity is less than 600 m/s, the static resistance can be calculated by Formula (10):

(10) $$R_{a}=97.1\times {\left(V/V_{R}\right)}^{p}f_{c}^{0.43}$$

where *V_R_* = 600 m/s, and the parameter *p* is a dimensionless parameter obtained by fitting, for ordinary concrete, *p* = 0.874.

**Figure 15:**
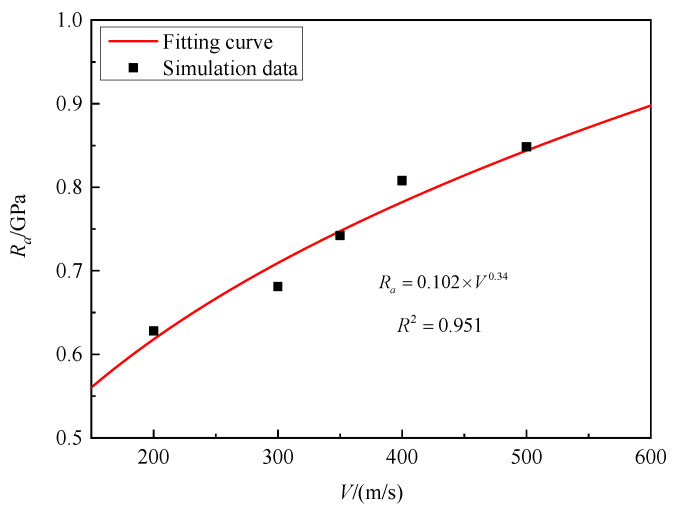
*R_a_* versus striking velocities for granite.

Figure 15 shows the static resistance of granite targets at impact velocities of 200 m/s, 300 m/s, 350 m/s, 400 m/s, and 500 m/s. The corresponding static resistances are 0.554 GPa, 0.681 GPa, 0.742 GPa, 0.807 GPa, and 0.848 GPa. In this sense, it can be concluded that static resistance significantly increases with increasing the striking velocity of projectiles. By fitting the numerical simulation data, the striking velocities parameter *ξ* = 0.34 was determined.

### 4.2. Effect of Projectile Mass

**Figure 16:**
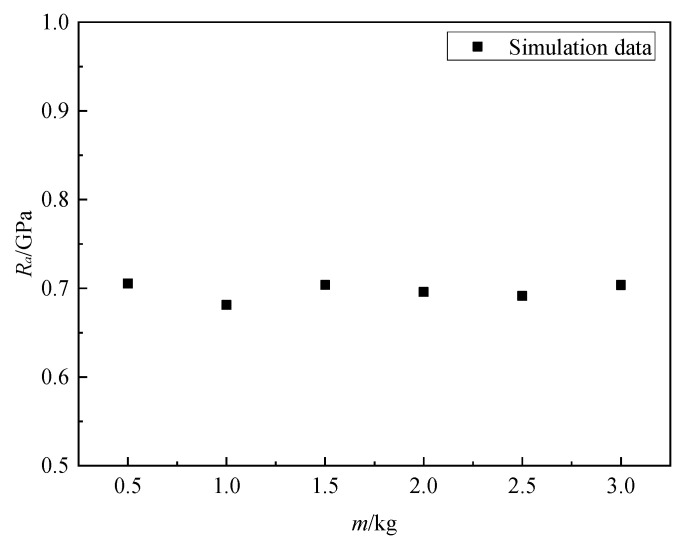
*R_a_* versus mass of the projectile for granite.

Yang [26] studied the impact response of a normal concrete target subjected to projectiles of different masses, and the results show that for the given concrete target, the static resistance was independent of the projectile mass. Figure 16 shows the static resistance of granite targets for projectiles with masses of 0.5 kg, 1.0 kg, 1.5 kg, 2.0 kg, 2.5 kg, and 3.0 kg at the impact velocity of 300 m/s are 0.705 GPa, 0.681 GPa, 0.704 GPa, 0.696 GPa, 0.691 GPa, and 0.704 GPa. It can be observed that the effect of projectile mass on static resistance is negligible.

### 4.3. Effect of CRH

**Figure 17:**
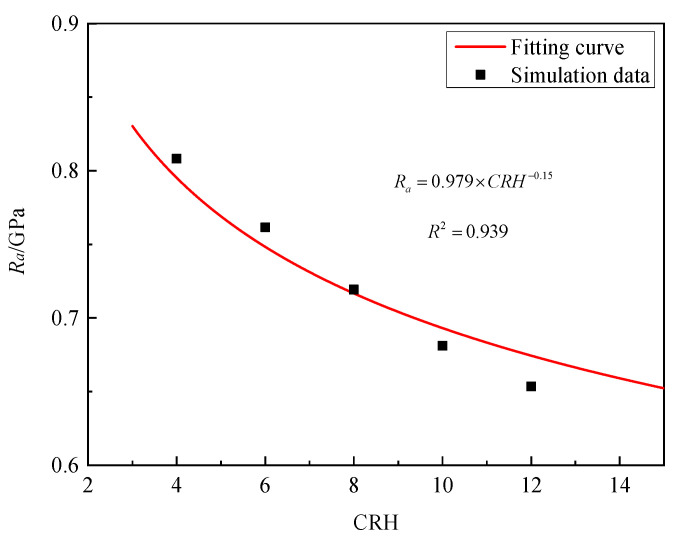
*R_a_* versus CRH of projectile for granite.

Wang’s research [27] suggests that the CRH of olive-nosed projectiles is one of the most important factors affecting static resistance. In order to ensure that the mass remains unchanged when the CRH changes, we achieve this goal by changing the density of the projectile. Figure 17 shows the static resistance of granite targets for a projectile with CRH of 4, 6, 8, 10, and 12 at the impact velocity of 300 m/s are 0.808, 0.762, 0.719, 0.681, and 0.653 GPa. This means that with the increase in CRH, the static resistance significantly decreases. By fitting the numerical simulation data, the CRH parameter *ϒ* = −0.15 was determined.

### 4.4. Effect of Projectile Diameter

**Figure 18:**
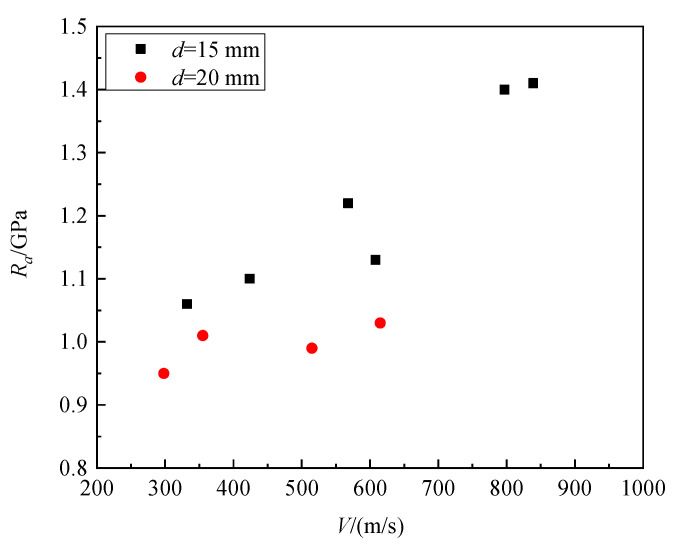
Comparison of static resistance of granite under the penetration of two projectiles.

Figure 18 shows the variation in the static resistance of granite under the penetration of two projectiles according to Ref [6]. It can be seen that the static resistance corresponding to the large-diameter projectile is significantly smaller than that of the small-diameter projectile.

Yang [26] found through numerical simulations that for projectiles of different diameters, the static resistance *R_a_* decreases with the increase in the projectile diameter; the static resistance can be calculated by Formula (11):

(11) $$R_{a}=117.88\times {\left(d/d_{0}\right)}^{\lambda }f_{c}^{0.43}$$

where *d*_0_ = 20.3 mm, and the parameter p is a dimensionless parameter obtained by fitting, for ordinary concrete, *λ* = −0.344

**Figure 19:**
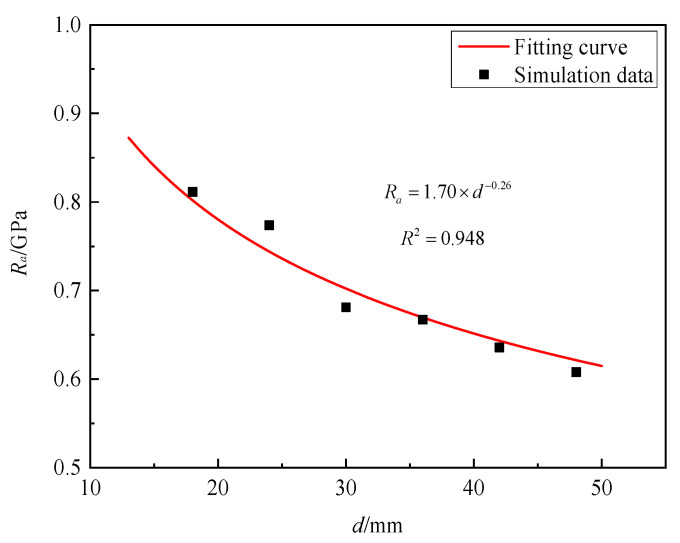
*R_a_* versus diameter of projectile for granite.

Figure 19 shows the static resistance of granite targets for projectiles with diameters of 18 mm, 24 mm, 30 mm, 36 mm, 42 mm, and 48 mm at the impact velocity of 300 m/s are 0.811 GPa, 0.773 GPa, 0.681 GPa, 0.667 GPa, 0.636 GPa, and 0.608 GPa. This means that with the increase in the projectile diameter, the static resistance gradually decreases. By fitting the numerical simulation data, the projectile diameter parameter *β* = −0.26 was determined.

### 4.5. Effect of Uniaxial Compressive Strength

**Figure 20:**
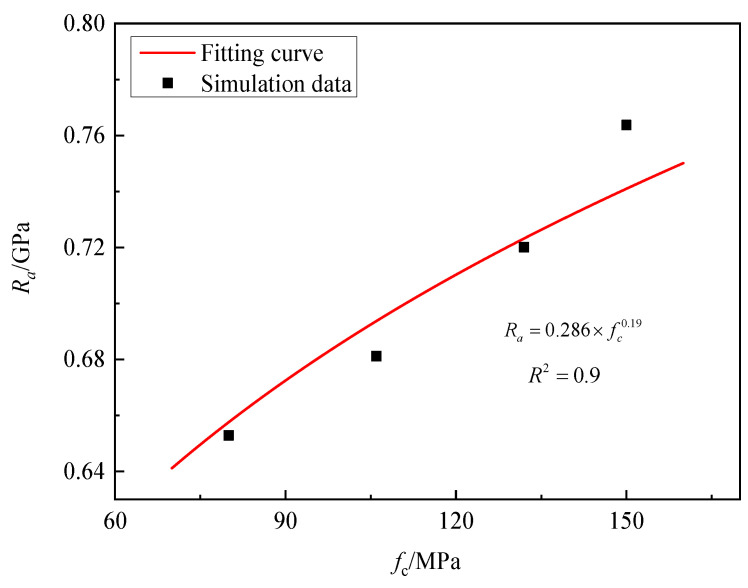
*R_a_* versus various compressive strengths of granite.

According to Figure 20, granite targets with 80 MPa, 106 MPa, 132 MPa, and 150 MPa static resistance were tested against projectile penetration with impact velocities of 300 m/s. It can be seen that as the compressive strength of the granite target increases, the static resistance gradually increases. When the compressive strength was 80 MPa, the corresponding static resistance was 0.653 GPa, while when the compressive strength increased to 150 MPa, the corresponding static resistance increased to 0.763 GPa. By fitting the numerical simulation data, the compressive strength parameter *λ* = 0.19 was determined.

### 4.6. Proposed Model to Predict Static Resistance

The least square method was used to fit the numerical simulation data to obtain the equation for predicting the static resistance of granite. The equation is as follows:

(12) $$R_{a}=1.66\times 10^{6}\cdot d^{-0.26}\cdot CRH^{-0.15}\cdot f_{c}^{0.19}\cdot V^{0.34}$$

where *R_a_* is the static resistance (Pa); CRH is the projectile caliber–radius–head ratio; *f*_c_ is the compressive strength of the granite target (Pa); *d* is the diameter of the projectile (m); and *V* is the projectile impact velocity (m/s).

**Figure 21:**
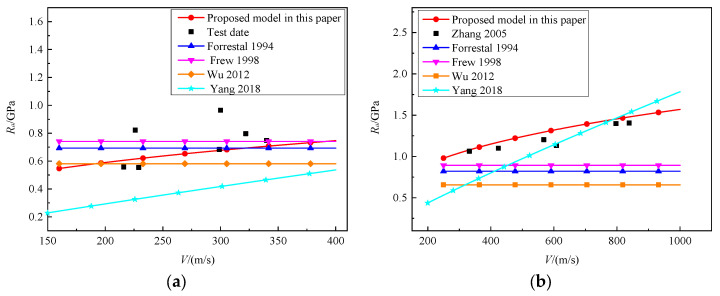
Test date and prediction models of R_a_ versus the impact velocities: (**a**) 106 MPa granite target [8,12,15,26]; (**b**) 154 MPa granite target [6,8,12,15,26].

We compared the formula for predicting static resistance proposed in this paper with the formula proposed by Forrestal [12], Frew [8], Wu [15], and Yang [26] shown in Figure 21. As can been seen in the figure, the influence of velocity is not considered in the Forrestal [12], Frew [8] and Wu [15] models for calculating static resistance, and there is no change in static resistance with the increase in velocity. Although Yang [26] considered the effect of velocity on static resistance, the prediction of the static resistance of granite is poor. When the velocity is below 600 m/s, the value of the static resistance is underestimated, while at higher velocities (V greater than 800 m/s), the value of the static resistance is overestimated. The empirical formula proposed in this paper not only considers the compressive strength of granite materials but also comprehensively considers the influence of striking velocities, projectile diameter, projectile mass, and CRH on the penetration static resistance, making it more consistent with the test results.

However, there are limitations in this study. Firstly, the formula proposed in this paper for predicting static resistance is only applicable to granite materials, and further analysis is needed to apply it to other target materials. Secondly, the effect of granite hardness on static resistance is not discussed in this paper, which is a problem that needs further study. Material hardness testing is relatively complex; Ref. [28] deals with the possibility of using the Schmidt hardness test, which does not require much preliminary preparation and is easy to perform in the production of commercial blocks in a quarry. Regarding the effect of material hardness on the static resistance of penetration, the authors will conduct a detailed discussion in future research.

## 5. Conclusions

In this study, 2 kinds of projectiles were used to penetrate a granite target at a speed of 216~340 m/s. The dimensionless penetration depth, dimensionless crater diameter, and mass loss rate of the projectiles were analyzed and compared. ANSYS 16.0/LS-DYNA finite element software was used to numerically simulate the process of projectile penetrating granite, and the influence of projectile and target parameters on static resistance *R_a_* was analyzed.

1. Two different types of projectiles were used to penetrate a granite target at the same speed. However, the dimensionless penetration depth of each projectile was different due to the size effect, with the larger projectile penetrating deeper than the smaller one. The mass loss rate also shows a similar size effect. The mass loss rate of the large projectile is smaller than that of the small projectile. It is possible that the size effect of the static penetration resistance contributed to the differences in dimensionless penetration depth and mass loss rate observed between the two projectiles.
2. The RHT model parameters of granite were determined, and the numerical model performed well in simulating penetration depth and crater damage.
3. To investigate the effect of the compressive strength of granite, striking velocities, projectile mass, diameter, and CRH on static resistance, parametric studies were conducted. An empirical formula to predict the static resistance *R_a_* was derived. Based on the parametric studies, an empirical formula was derived to predict the static resistance *R_a_*.

## Figures and Tables

**Table 1:** Basic parameters of the projectiles.

Order Number	Diameter of the Projectile (mm)	Weight of the Projectile (kg)	Length of the Projectile (mm)	CRH
Projectile A	30	1.0	307	10
Projectile B	78	17.5	800	10

**Table 2:** Mechanical parameters of granite.

Elastic Modulus of Granite (GPa)	Uniaxial Compressive Strength of Granite (MPa)	Density of Granite (kg·m^−3^)	Poisson’s Ratio of Granite	Tensile Strength of Granite (MPa)
48.5	106	2600	0.17	6.5

**Table 3:** Penetration tests data.

Test No	Targets NO	*d* ^1^ (mm)	*m* ^2^ (kg)	*V* ^3^ (m/s)	Depth of Penetration		Crater Diameter (mm)	Mass Loss	Static Resistance (GPa)
					*h* (mm)	*h*/*d*			
1	gr-1	30	0.999	216	89	2.97	253	1.1%	0.557
2	gr-2	30	1.002	226	74	2.47	370	1.3%	0.822
3	gr-3	30	1.003	229	97	3.23	305	1.3%	0.554
4	gr-4	30	0.999	300	122	4.07	363	1.9%	0.683
5	gr-5	30	1.004	300	96	3.2	365	2.0%	0.965
6	gr-6	30	1.005	322	122	4.07	513	2.4%	0.797
7	gr-7	30	1.003	340	139	4.6	363	2.7%	0.748
8	gr-8	78	16.823	310	420	5.38	860	0.9%	0.491
9	gr-9	78	16.985	320	460	5.90	900	0.6%	0.473

^1^ Projectile diameter; ^2^ projectile mass; and ^3^ striking velocities.

**Table 4:** RHT model parameters for granite.

ρ (kg·m^−3^)	*G* (GPa)	$f_{c}$ (MPa)	$B_{1}$	$B_{2}$	$T_{1}$ (GPa)	$T_{2}$	*A*	${\dot{\varepsilon }}^{c}$ (s^−1^)	${\dot{\varepsilon }}^{t}$ (s^−1^)	${\dot{\varepsilon }}_{0}^{c}$ (s^−1^)	${\dot{\varepsilon }}_{0}^{t}$ (s^−1^)
2600	22	106	0.732	0.732	77.2	0	2.286	3.0 × 10^25^	3.0 × 10^25^	3.0 × 10^−5^	3.0 × 10^−6^
$p_{el}$ (MPa)	$g_{c}^{*}$	$g_{t}^{*}$	ξ	$D_{1}$	$\varepsilon_{p}^{m}$	$A_{f}$	$n_{f}$	$A_{1}$ (GPa)	$A_{2}$ (GPa)	$A_{3}$ (GPa)	$\beta_{c}$
35.3	0.53	0.7	0.67	0.04	0.008	1.75	0.52	77.2	56.5	16.5	0.0118
$\beta_{t}$	*B*	*N*	$D_{2}$	$Q_{0}$	*n*	$f_{t}^{*}$	$f_{s}^{*}$	$p_{\mathrm{com}}$(GPa)	$\alpha_{0}$		
0.0159	0.0105	4.0	1	0.681	0.61	0.061	0.267	6	1.18		

ρ is the density; *G* is the shear modulus; $f_{c}$ is the compressive strength; $B_{1}$, $B_{2}$, $T_{1}$, $T_{2}$ are the parameters for polynomial EOS; $A_{1}$, $A_{2}$, $A_{3}$ are the hugoniot polynomial coefficients; $f_{t}^{*}$ is the relative tensile strength; $f_{s}^{*}$ is the relative shear strength; *A*, *N* are the failure surface parameters; $Q_{0}$, *B* are the lode angle dependence factors; *N* is the porosity exponent; ${\dot{\varepsilon }}_{0}^{c}$ is the reference compressive strain rate; ${\dot{\varepsilon }}_{0}^{t}$ is the reference tensile strain rate; ${\dot{\varepsilon }}^{c}$ is the break compressive strain rate; ${\dot{\varepsilon }}^{t}$ is the break tensile strain rate; $\beta_{c}$ is the compressive strain rate dependence exponent; $\beta_{t}$ is the tensile strain rate dependence exponent; $g_{c}^{*}$ is the compressive yield surface parameter; $\varepsilon_{p}^{m}$ is the minimum damaged residual strain; ξ is the shear modulus reduction factor; $D_{1}$, $D_{2}$ are the damage parameters; $A_{f}$, $n_{f}$ are the residual surface parameters; *A*, *n* are the failure surface parameters; $\alpha_{0}$ is the initial porosity; $p_{el}$ is the crush pressure; and $p_{\mathrm{com}}$ is the compaction pressure.

**Table 5:** Model parameters for the projectile.

ρ (kg·m^−3^)	*E* (GPa)	$\sigma_{Y}$ (MPa)	ν	$E_{t}$ (GPa)	β	*C*	*P*	$F_{s}$
7800	210	1300	0.3	9	1	40	5	0.2

ρ is the density; *E* is the elastic modulus; $\sigma_{Y}$ is the yield strength; ν is Poisson’s ratio; $E_{t}$ is the tangent modulus; β is the hardening parameters; *C*, *P* is the strain rate parameter, and $F_{s}$ is the failure strain.

**Table 6:** Comparison of experimental and numerical penetration depth.

Target No	Diameter of the Projectile (mm)	Velocities *V* (m/s)	Experiments (mm)	Simulation (mm)	Error
gr-1	30	216	89	82	7.9%
gr-4	30	300	122	123	0.8%
gr-7	30	340	139	143	2.9%
gr-8	78	310	420	382	9.5%

**Table 7:** Parametric studies on granite targets against projectile penetration.

Test No	Compressive Strength *f*_c_ (MPa)	CRH	Velocities *V* (m/s)	Mass *m* (kg)	Diameter *d* (mm)	DOP (mm)	Static Resistance *R_a_*
1	106	10	200	1.0	30	81	0.554
2	106	10	300	1.0	30	123	0.681
3	106	10	350	1.0	30	146	0.742
4	106	10	400	1.0	30	169	0.807
6	106	10	500	1.0	30	236	0.848
9	106	10	300	0.5	30	75	0.705
10	106	10	300	1.0	30	123	0.681
11	106	10	300	1.5	30	165	0.704
12	106	10	300	2.0	30	212	0.696
13	106	10	300	2.5	30	259	0.691
14	106	10	300	3.0	30	300	0.704
15	106	4	300	1.0	30	108	0.808
16	106	6	300	1.0	30	113	0.762
17	106	8	300	1.0	30	118	0.719
18	106	10	300	1.0	30	123	0.681
19	106	12	300	1.0	30	127	0.653
20	106	10	300	1.0	18	235	0.811
21	106	10	300	1.0	24	152	0.773
22	106	10	300	1.0	30	123	0.681
23	106	10	300	1.0	36	102	0.667
24	106	10	300	1.0	42	93	0.636
25	106	10	300	1.0	48	89	0.608
26	80	10	300	1.0	30	127	0.653
27	132	10	300	1.0	30	118	0.720
28	150	10	300	1.0	30	113	0.763

## Data Availability

The data presented in this study are available on request from the corresponding author.
